# RETRACTION: Xiao Yao San against Corticosterone‐Induced Stress Injury via Upregulating Glucocorticoid Receptor Reaction Element Transcriptional Activity

**DOI:** 10.1155/ecam/9780460

**Published:** 2026-02-12

**Authors:** Evidence-Based Complementary and Alternative Medicine

RETRACTION: G. Cao, S. Gong, F. Zhang, and W. Fu, “Xiao Yao San against Corticosterone‐Induced Stress Injury via Upregulating Glucocorticoid Receptor Reaction Element Transcriptional Activity,” *Evidence-Based Complementary and Alternative Medicine*, no. 2016 (2016). https://doi.org/10.1155/2016/5850739.

The above article, published online on 16 October 2016 in Wiley Online Library (https://wileyonlinelibrary.com), has been retracted by John Wiley & Sons Ltd.

The retraction has been agreed following an investigation of the concerns raised by *Otiophora multicaulis* and *Pothos tener* on PubPeer [1], which identified several instances of overlapping features in multiple figures of the manuscript:

- Figure 1c: All images of the MAP‐2 immunopositive neurons contain overlapping features.
- Figure 2b: The cell cycle analysis graphs of the “Low” and “Medium” groups are unexpectedly similar.

As a result of the investigation, the data and conclusions of this article are considered unreliable.

The authors have been informed of this retraction but did not provide a response.
